# Intron and gene size expansion during nervous system evolution

**DOI:** 10.1186/s12864-020-6760-4

**Published:** 2020-05-14

**Authors:** Matthew J. McCoy, Andrew Z. Fire

**Affiliations:** 1 Grass Fellowship Program, Marine Biological Laboratory, Woods Hole, MA 02543 USA; 2 Departments of Pathology and Genetics, Stanford University School of Medicine, Stanford, CA 94305 USA

**Keywords:** Genome evolution, Gene size, Intron size, Nervous system evolution, Long genes, Long introns

## Abstract

**Background:**

The evolutionary radiation of animals was accompanied by extensive expansion of gene and genome sizes, increased isoform diversity, and complexity of regulation.

**Results:**

Here we show that the longest genes are enriched for expression in neuronal tissues of diverse vertebrates and of invertebrates. Additionally, we show that neuronal gene size expansion occurred predominantly through net gains in intron size, with a positional bias toward the 5′ end of each gene.

**Conclusions:**

We find that intron and gene size expansion is a feature of many genes whose expression is enriched in nervous systems. We speculate that unique attributes of neurons may subject neuronal genes to evolutionary forces favoring net size expansion. This process could be associated with tissue-specific constraints on gene function and/or the evolution of increasingly complex gene regulation in nervous systems.

## Background

Genome size and complexity greatly increase from prokaryotes to multicellular eukaryotes [1]. The changes include increases in the size and number of introns, in large part through dramatic proliferation and accumulation of mobile genetic elements [2–4] and through shifts in the balance of deletions and insertions [5]. The differential expansion of intronic sequences has led to orders of magnitude variation in overall gene lengths within genomes and among species across diverse phylogenetic lineages (see Additional file 1: Figure S1 for an example in which gene size and density vary dramatically among animal orthologs of *NRXN1*, which encode cell-surface receptors localized at neuronal synapses).

The evolutionary consequences of gene size expansion are still being uncovered. Although the causes and consequences may be intertwined, both are areas of valuable investigation. Longer genes tend to have more splice variants [6], which give rise to alternative isoforms with distinct molecular functions and expression patterns. Additionally, long genes can have greater numbers of regulatory elements, with these arrayed in more diverse patterns [7]. Over 10% of human protein-coding genes are greater than 100 kb in length, with the time required to transcribe these long genes being significant on a cellular, developmental and organismal level [8]. Although exact transcription rates are unknown, assuming the estimated average rates of transcription in eukaryotes of 1–4 kb per minute [9, 10], the 2.3 Mb human gene *CNTNAP2* would require upwards of 10 h to generate one transcript, which is substantially longer than the 10 min required for the average mammalian gene ~ 10 kb in length [11]. This phenomenon, termed intron delay, biases patterns of expression and evolution of gene structure for a substantial portion of the transcriptome [12, 13]. We hypothesize that this potentially limits expression of long genes to long-lived post-mitotic cells, such as neurons. Supporting this, recent transcriptomic studies in rodents and humans have revealed that expression of the longest genes is enriched in neuronal tissues [7, 14–17]. Additionally, many of these genes encode ion channels, receptors, and cell adhesion molecules [15], which greatly contribute to the extensive molecular diversity that exists among neuronal subtypes within rodents and humans [7, 17, 69]. Finally, a role for long genes in genetic causation of cancer and neurological disease is becoming apparent [14, 15, 70, 71], potentially from elevated vulnerability to mutation [70, 71]. Using evidence from more extensive phylogenomics and transcriptomics, we show here that long gene expression enrichment in neuronal tissues is a phenomenon extending beyond rodents and humans. Additionally, we show that net expansion of neuronal intron and gene size is a feature of many nervous systems.

## Results

**Fig. 1:**
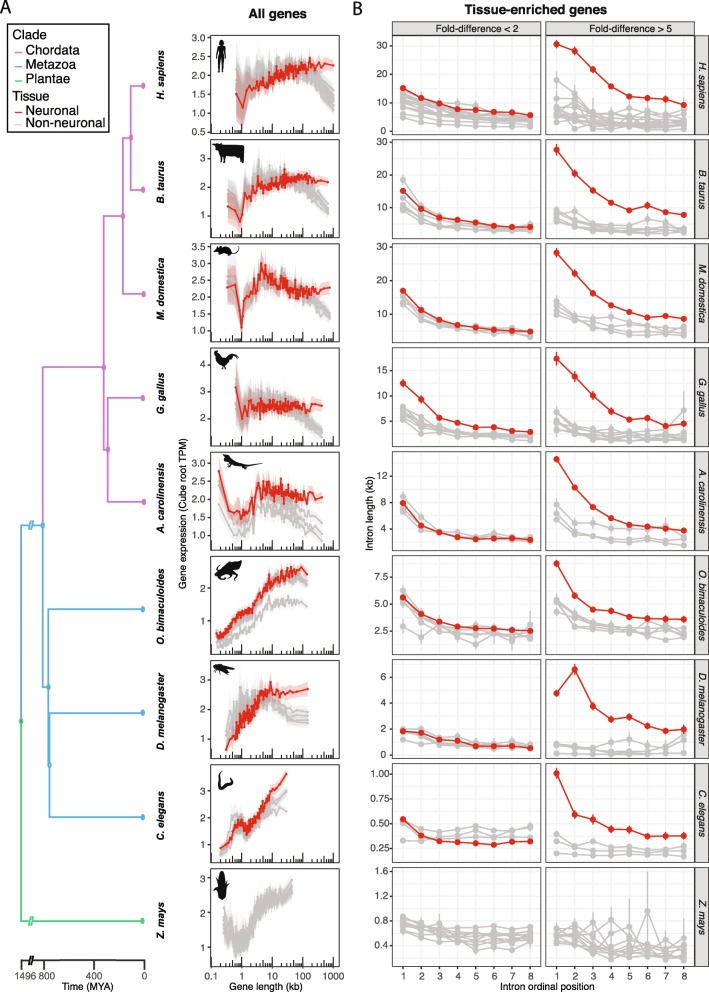
Neuronal intron and gene size expansion in diverse animal species. **a** Expression of the longest genes is enriched in neuronal tissues of vertebrates and invertebrates. Each plot shows smooth-quantile-normalized transcriptome data across multiple tissues or cell types from individual species. Each line represents mean gene expression in cube root transcripts per million (TPM) versus gene length (kb). Genes were segregated into 100 bins according to gene length, and points show average gene length of each bin. Transparent ribbons show 95% confidence intervals. Red lines show neuronal tissues and grey lines show non-neuronal tissues (see Additional file 2: Table S1 for a full list of tissues for each species). Tissue transcriptomes were obtained from the EMBL Expression Atlas [21] for all species except *Drosophila melanogaster* [18] and *Octopus bimaculoides* [19]. Branch lengths for the phylogenetic tree were obtained from TimeTree.org [22]. Organism outlines were depicted by author MJM from the following image references in accordance with respective licensing: *Homo sapiens* [23]; *Bos taurus* [24]; *Monodelphis domestica* [25]; *Gallus gallus* [26]; *Anolis carolinensis* [27]; *Octopus bimaculoides* [28]; *Drosophila melanogaster* [29]; *Caenorhabditis elegans* [30]; and *Zea mays* [31]. **b** Mean intron length (kb) versus ordinal position in neuronal (red) and non-neuronal tissues (grey) across the same species presented in Fig. 1a. Genes with only slight expression fold-difference of the top expressing tissue over the next highest-expressing tissue (< 2 fold-difference; left) are contrasted against genes with more than 5 fold-difference in expression (right). Error bars show standard error

Enrichment of long gene expression in neuronal tissues has previously been observed in rodents and humans [7, 14–17]. By analyzing systems with well-defined tissue transcriptomes [18–20], we asked whether neuronal long gene expression is a feature shared across diverse taxonomic classes both within the chordate phylum and among invertebrates. We examined gene expression as a function of gene length (see Gene Expression Analysis in Methods) in multiple tissues for diverse animals and found that as gene length increases, so does expression enrichment in neuronal tissues (Fig. 1a).

Neuronal long gene expression is also apparent in invertebrates, such as the insect arthropod, *Drosophila melanogaster*, and the cephalopod mollusc, *Octopus bimaculoides* (Fig. 1a). As observed in comparing read counts in different tissues with annotated gene lengths, both assembled genomes exhibit enrichment for neuronal transcripts in size ranges at the high end of the gene-length distribution (e.g. the top ~ 14% of genes in *D. melanogaster,* which are > 10 kb, and the top ~ 14% of genes in *O. bimaculoides*, which are > 30 kb). Cephalopods are particularly interesting in part because they have the largest known invertebrate nervous systems [32], along with sophisticated behaviors [33]. Cephalopods are estimated to have shared their most recent common ancestor with insects more than 750 million years ago [22], which is only ~ 50 million years later than the estimated pairwise divergence between vertebrates and invertebrates [22]. Together, these observations support either an ancient origin of neuronal long gene preference dating back to the evolution of the first nervous systems or convergence toward this phenomenon in multiple animal lineages.

Within eukaryotic genes, individual intron lengths negatively correlate with intron ordinal position [34, 35], with 5′ introns being larger and containing more conserved sequences relative to downstream introns [36, 37]. Here we analyzed intron length versus intron ordinal position for genes enriched in different tissues. To calculate a rough metric for tissue-specificity, we stratified genes based on maximal expression in each tissue, then calculated fold-difference in expression over the next-highest expressing tissue. In genes for which expression was substantially enriched in neuronal tissues (> 5-fold, Fig. 1b), we observed striking enrichment for long introns, particularly near the 5′ ends of genes. This enrichment declines toward the 3′ ends of genes, and in genes with lesser neuronal expression enrichment (< 2-fold, Fig. 1b; > 2-fold, Additional file 3: Figure S2).

The rapidly developing invertebrate nematode, *Caenorhabditis elegans* has a very compact genome and compact gene sizes (Fig. 1a). Nonetheless, indication of longer introns in neuronally expressed *C. elegans* genes is evident, as is some indication of neuronal enrichment in the longest genes (Fig. 1a; see Additional file 4: Figures S3, Additional file 5: Figures S4, Additional file 6: Figures S5, Additional file 7: Figures S6 for alternative normalization and plotting; see Gene Expression Analysis in Methods for further discussion). A detailed tissue-specific intron and gene length analysis was also possible for *Zea mays* (Fig. 1), showing compact genes and no evident expression enrichment for the longest genes in any tissues.

**Fig. 2:**
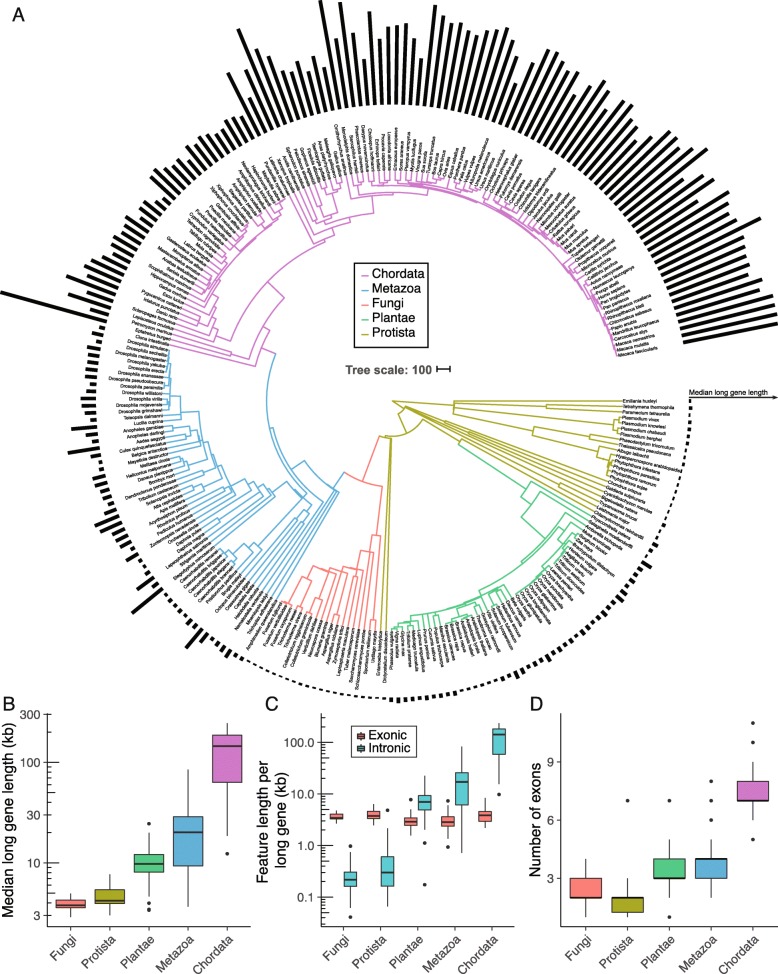
Differential gene size expansion during animal evolution. **a** Phylogenomic tree showing median gene length of the top 10% longest genes in each genome as a bargraph. All eukaryotic kingdoms are represented, with Metazoa further subdivided into animals with notochords (Chordata) and animals without notochords (here labeled as Metazoa). Branch lengths were obtained from TimeTree.org [22], and scale bar shows 100 million years. **b** Boxplots of median gene length of long genes of each genome in kilobases aggregated by clade. **c** Boxplots of long gene intronic sequence versus exonic sequence per clade. **d** Boxplots showing median number of exons of each gene for each species grouped by clade. This extends similar analyses by Lynch et al. [40] and Francis and Wörheide [39] of exon/intron content

Large 5′ introns and the overall length increases of genes enriched in neuronal tissues of both vertebrates and invertebrates may be linked to broader trends in animal genome evolution. For example, although many orthologs of long neuronal genes are relatively long in other animals, gene size and density are strikingly different between vertebrates and invertebrates (Additional file 1: Figure S1). To highlight these differences, we compared protein-coding gene lengths across 325 eukaryotic species currently available on the Ensembl genome database [38]. We chose a measurement of gene length spanning the start of the first exon to the end of the last exon, in part to avoid known differences in annotation completeness of untranslated regions (UTRs) [39]. To compare gene lengths across eukaryotic species, we calculated the median gene lengths within each species (Fig. 2a), including in Protista ($\overline{x}$ = 1.36 ± 0.33 kb [standard deviation]), Plantae ($\overline{x}$ = 2.42 ± 0.71 kb), Fungi ($\overline{x}$ = 1.46 ± 0.26 kb), Metazoa ($\overline{x}$ = 2.79 ± 1.71 kb; excluding chordates), and Chordata ($\overline{x}$ = 12.88 ± 5.43 kb). Gene length variation was most dramatic for the top 10% longest genes, which were an order of magnitude longer in vertebrates ($\overline{x}$ = 129.23 ± 64.12 kb) compared to non-animal eukaryotes ($\overline{x}$ = 7.29 ± 4.26 kb)(Fig. 2b). Consistent with other reports [39, 40], the vast majority of the increases in gene length are intronic (Fig. 2c), with increases in intron length coinciding with increases in the average number of exons per gene (Fig. 2d; Additional file 8: Figure S7). Within Metazoa, multicellular animals lacking nervous systems (e.g. placazoa and sponges) had significantly shorter genes than most animals possessing nervous systems (*p* < 2.2e-16, wilcoxon signed rank test with continuity correction), even when only considering invertebrates (*p* = 4.0e-10) (Fig. 2a). Many of the observed differences were consistent within species of the same clade, some of which include more extensively annotated organisms (e.g. *Homo sapiens*, *Drosophila melanogaster*, *Arabidopsis thaliana*, etc.), but since annotation quality and completeness are continually improving [39], the exact values presented here for individual species are expected to change in the future. Although the evolutionary dynamics of gene size expansion alone do not explain how the longest genes became enriched for neuronal expression in particular, the observed median sizes are consistent with metazoan gene size expansion beginning during the evolutionary divergence of animals in the Precambrian eon [41]. Together with evidence of neuronal long gene expression in vertebrates and invertebrates, this suggests that intron and gene size expansion of neuronal genes could have begun during the evolution of the first nervous systems.

## Discussion

The evolutionary course of intron and gene size net expansion or net contraction is expected to be a combined function of insertion/deletion frequencies, of selective pressure, and of neutral processes such as genetic drift. Determining the individual and collective contribution of these forces is not trivial, with a primary challenge coming from the currently necessary use of static distributions of gene sizes across Eukarya as presented here. Nonetheless, certain features emerge from the analysis even with the static picture. Our observation that 5′ end introns of neuronal genes are longer than in genes enriched in other tissues is one such feature, potentially indicating differences between tissues that influence tolerance for insertion/deletion events. For eukaryotic genes in general, intron length bias near 5′ ends of genes is generally thought to emerge over evolutionary time from a shift in the insertion/deletion spectrum in favor of insertions, since longer deletions more frequently disrupt functional elements that are enriched near 5′ ends of genes [36, 37, 72].

Regardless of how introns undergo net size expansion, as introns lengthen, they have higher probabilities of gaining novel exons, which contribute to alternative isoform diversity [42, 43]. Furthermore, new exon-containing isoforms are typically rare, which potentiates the generation of novel functions with minimal damage to the existing functional repertoire [44]. These previous observations have contributed to hypotheses regarding the role of alternative splicing in the evolution of overall phenotypic complexity [45, 46]. We speculate that intron and gene size expansion parallels functional element complexity of neuronal genes, and that size expansion may potentiate further increases in gene regulatory complexity and molecular diversity of nervous systems.

There are several outliers for gene and genome size among animals. The microbat (*Myotis lucifugus*) has one of the smallest mammalian genomes at ~ 2 Gb, likely from extensive DNA loss [47] and correspondingly smaller gene sizes (Fig. 2a). There is some evidence that the metabolic cost of powered flight is associated with smaller genomes [48], and that the smaller sizes observed in bats and birds results from DNA loss through segmental deletions rather than decreased repetitive element expansion [47]. In other seeming exceptions, reduced gene sizes could potentially arise from technical limitations as opposed to bona fide biological differences. One possible example of this is the American black bear (*Ursus americanus*) whose genome assembly has low contiguity (contig N50 = 27,247 bp; scaffold N50 = 189,900 bp) [49], which could limit gene annotation completeness. Certain fish also exhibit compact gene and genome sizes, such as the teleosts which underwent an additional round of whole-genome duplication approximately 320–400 million years ago [50]. Genes with larger gene families tend to have shorter genes [6], which has been proposed to result from the partial nature of duplications [51] as longer genes undergo incomplete duplication more frequently than shorter genes. However, in the context of duplicated whole-genomes, smaller gene sizes may reflect reduced selective pressure to maintain functional sequences within introns of a duplicated gene. Conversely, hagfish (*Eptatretus burgeri*) have one of the larger median gene lengths (Fig. 2a). Both hagfish and lamprey are jawless vertebrates and the only known chordates to undergo dramatic remodeling of their genome, eliminating hundreds of millions of base pairs from many somatic cells during embryonic development [52]. It remains unknown how these extreme rearrangements contribute to gene and genome size evolution. While these examples highlight several parameters potentially affecting the dynamics of gene and genome size evolution at the organismal level, our study indicates that biological parameters at the tissue and cellular level may also shape aspects of gene and genome architecture.

## Conclusions

The molecular components of nervous systems are similar across animals despite vast differences in nervous system complexity, with some of the same sets of proteins and signaling molecules being used repeatedly in different contexts [53]. Intron and gene size expansion provide one mechanism by which existing components can be modified to increase transcriptomic potential from a limited gene complement, contributing to the evolution of novelty [6, 54] and potentially to the molecular complexity that is a hallmark of nervous systems. In this study, we show that many of the longest genes are preferentially expressed in neuronal tissues across diverse vertebrates and invertebrates. Additionally, we find longer introns in genes with neuronal expression enrichment, with introns near the 5′ ends of such genes being particularly lengthy. Determining the degree to which intron and gene size expansion are under the influence of adaptive and/or non-adaptive forces will be critical to understanding their evolutionary trajectory and phenotypic consequences. We hypothesize that intron and gene size expansion is uniquely tolerated, or perhaps uniquely advantageous in neuronal genes, and that expansion may enable the acquisition of new exons, alternative isoforms, and enhancers active within nervous tissue.

## Methods

### Statistical analysis

All statistical tests were performed in R version 3.5.0 (R Core Team 2018) and RStudio version 1.1.453 (RStudio Team 2015). All analyses are available as R scripts accompanied by data tables.

### *NRNX1* Ortholog display

Orthologs of the human *NRXN1* gene were obtained by using the Drosophila RNAi Screening Center Integrative Ortholog Prediction Tool (DIOPT, version 8.0) [55], which integrates ortholog predictions from Ensembl Compara, HomoloGene, Inparanoid, Isobase, OMA, orthoMCL, Phylome, RoundUp, and TreeFam. The highest scoring reciprocal hits were selected for display.

### Gene expression analysis

RNA-seq data were obtained from NCBI’s Sequence Read Archive (SRA) for *O. bimaculoides* (PRJNA285380) [19] and *D. melanogaster* (SRR070409, SRR070410, SRR070408, SRR070405, SRR070406, SRR070392, SRR070393, SRR070407, SRR070425, SRR100268) [18]. *O. bimaculoides* reads was aligned to *Octopus bimaculoides* v2_0 reference genome and *D. melanogaster* reads to *Drosophila melanogaster* Release 6 reference genome using the R package ‘Rsubread’ [56], and read counts were normalized to TPM. All other transcriptomes were obtained as TPM-normalized counts from the EMBL Expression Atlas [21]: *H. sapiens* (E-MTAB-513) [57], *B. taurus* (E-MTAB-2596) [58], *M. domestica* (E-MTAB-3719) [59], *G. gallus* (E-MTAB-2797) [60], *A. carolinensis* (E-MTAB-3727) [57], *C. elegans* (E-MTAB-2812) [61], and *Z. mays* (E-GEOD-62778) [62].

Smoothed conditional means of gene expression conditional on gene length (or gene length percentiles) were estimated using the ‘geom_smooth’ function from R package ggplot2 version 2.2.1 [63]. Two different smoothing methods were used: ‘gam’, which is a generalized additive model with formula = y ~ s(x, bs = “cs”) using the restricted maximum likelihood (REML) approach for smoothing parameter estimation; and ‘loess’, which is a local polynomial regression fitting with formula = y ~ x and span (neighborhood) of proportion 0.1. To visualize the data without the underlying assumptions of smoothing across gene lengths varying by several orders of magnitude, we also (i) analyzed gene expression across gene length percentiles and observed a similar expression enrichment in neuronal tissues for the longest genes (Additional file 5: Figures S4, Additional file 6: Figures S5, Additional file 7: Figures S6), and (ii) analyzed mean gene expression of genes binned into 100 bins (Fig. 1a; Additional file 4: Figures S3, Additional file 5: Figures S4, Additional file 7: Figures S6). In addition to TPM normalization, data in Fig. 1a and Additional file 6: Figures S5, Additional file 7: Figures S6 were subjected to a generalization of quantile normalization called smooth quantile normalization (function ‘qsmooth’ from R package qsmooth version 1.2.0), which is based on the assumption that the statistical distribution of each sample should be the same within biological groups (e.g. tissue type), but allowing that they may differ between groups [64]. All analytical pipelines gave essentially the same result for gene expression versus gene length, with the possible exception of *C. elegans*.

For tissue-enrichment analyses (Fig. 1b), genes were only considered tissue-enriched if their expression value in their maximal tissue exceeded 50 times the lowest value for that tissue.

### Gene architecture quantification

Gene lengths in each species were obtained from Ensembl BioMart version 2.40.4 [38]. Gene start positions were subtracted from gene end positions to obtain a measure of gene length for each gene that excludes 5′ and 3′ UTRs. Protein coding genes were selected using gene biotype information. Human orthologs were also obtained from Ensembl BioMart [38]. Exon numbers were estimated after determining the union of known exon-coding sequences (Fig. 2d), or by calculating the average number of exons across all transcripts of each gene (Additional file 8: Figure S7). Intron lengths were calculated from exon positions of each transcript of each gene. Exonic and intronic content were determined from the union of all annotated exon and intron sequences for each isoform of each gene, respectively (Fig. 2c). Protein-coding gene lengths for each clade were reported in the main text first by obtaining the median gene lengths of either all genes or only the top 10% longest genes in each genome, then obtaining the mean and standard deviation of these values across each clade. Significant differences in gene lengths between a species and all other species of a clade were determined by wilcoxon signed rank test with continuity correction.

### Quantification of divergence

The branch lengths obtained from TimeTree.org [22] were combined with gene length annotations using the R packages ape version 5.1 [65] and phylobase version 0.8.4 [66]. Data was visualized using the R packages ggplot2 version 2.2.1 [63] and RColorBrewer version 1.1–2 [67], as well as using the Interactive Tree of Life (itol.embl.de) [68].

## Supplementary information

**Additional file 1: Figure S1.** An exemplary gene size and density comparison at *NRXN1* ortholog loci. For each species, a *NRXN1* ortholog (black; *NRXN1* in *Homo sapiens*, *Nrxn1* in *Mus musculus*, and *nrx-1* in *Drosophila melanogaster* and *Caenorhabditis elegans*) is shown centrally, while other genes (random non-black colors) are jittered vertically while maintaining their linear position in the genome. Each locus is shown in the context of a 10 million bp window, with one additional inset for *C. elegans* showing a 200 kb window surrounding *nrx-1*.

**Additional file 2: Table S1.** Tissues analyzed in each species of Fig. 1.

**Additional file 3: Figure S2.** Intron length versus ordinal position for genes with greater than 2-fold tissue enrichment.

**Additional file 4: Figure S3.** Non-smooth-quantile-normalized gene expression conditional on gene length. Each plot shows transcriptome data across multiple tissues from species presented in Fig. 1. Neuronal tissues or cells are shown in red and all other tissues and cell types are shown in grey, and transparent ribbons show 95% confidence intervals. (**A**) Genes were segregated into 100 bins according to gene length (kb), and points show average gene length of each bin. (**B**) Loess smoothing of gene expression conditional on gene length (kb). (**C**) Generalized additive model of gene expression conditional on gene length (kb).

**Additional file 5: Figure S4.** Non-smooth-quantile-normalized gene expression conditional on gene length quantiles. Each plot shows transcriptome data across multiple tissues from species presented in Fig. 1. Neuronal tissues or cells are shown in red and all other tissues and cell types are shown in grey, and transparent ribbons show 95% confidence intervals. Gene lengths are shown as quantiles (100 bins). (**A**) Genes were segregated into 100 bins according to gene length, and points show average gene length of each bin. (**B**) Loess smoothing of gene expression conditional on gene length quantiles. (**C**) Generalized additive model of gene expression conditional on gene length quantiles.

**Additional file 6: Figure S5.** Smooth-quantile-normalized gene expression conditional on gene length. Each plot shows transcriptome data across multiple tissues from species presented in Fig. 1. Neuronal tissues or cells are shown in red and all other tissues and cell types are shown in grey, and transparent ribbons show 95% confidence intervals. (**A**) Loess smoothing of gene expression conditional on gene length (kb). (**B**) Generalized additive model of gene expression conditional on gene length (kb).

**Additional file 7: Figure S6.** Smooth-quantile-normalized gene expression conditional on gene length quantiles. Each plot shows transcriptome data across multiple tissues from species presented in Fig. 1. Neuronal tissues or cells are shown in red and all other tissues and cell types are shown in grey, and transparent ribbons show 95% confidence intervals. Gene lengths are shown as quantiles (100 bins). (**A**) Genes were segregated into 100 bins according to gene length, and points show average gene length of each bin. (**B**) Loess smoothing of gene expression conditional on gene length quantiles. (**C**) Generalized additive model of gene expression conditional on gene length quantiles.

**Additional file 8: Figure S7.** Gene features across clades. Joy plots showing distribution of gene features (median gene length, median intron length, median exon length, median number of exons per gene, and number of genes).

## Data Availability

Transcriptome data for *Octopus bimaculoides* was obtained from SRA BioProject PRJNA285380 [19]. Transcriptome data for *D. melanogaster* was obtained from SRA (SRR070409, SRR070410, SRR070408, SRR070405, SRR070406, SRR070392, SRR070393, SRR070407, SRR070425, SRR100268) [18]. All other transcriptomes were obtained as TPM-normalized counts from the EMBL Expression Atlas: *H. sapiens* (E-MTAB-513) [57], *B. taurus* (E-MTAB-2596) [58], *M. domestica* (E-MTAB-3719) [59], *G. gallus* (E-MTAB-2797) [60], *A. carolinensis* (E-MTAB-3727) [57], *C. elegans* (E-MTAB-2812) [61], and *Z. mays* (E-GEOD-62778) [62]. The datasets generated and analyzed for the current study are available in the GitHub repository, https://github.com/mjmccoy/BMC_Genomics_2020

## Abbreviations

bp: Base pair
DNA: Deoxyribonucleic acid
GAM: Generalized additive model
Gb: Gigabase
kb: Kilobase
LOESS: Locally estimated scatterplot smoothing
MYA: Million years ago
REML: Restricted maximum likelihood
SRA: Sequence read archive
TPM: Transcripts per million
UTR: Untranslated region
