# Supplementary material for: Intron and gene size expansion during nervous system evolution
Source: BMC Genomics. 2020 May 14;21:360. doi: 10.1186/s12864-020-6760-4 (PMC7222433; doi:10.1186/s12864-020-6760-4)
Supplement: Supplementary file 1 — Additional file 1: Figure S1. An exemplary gene size and density comparison at NRXN1 ortholog loci. For each species, a NRXN1 ortholog (black; NRXN1 in Homo sapiens, Nrxn1 in Mus musculus, and nrx-1 in Drosophila melanogaster and Caenorhabditis elegans) is shown centrally, while other genes (random non-black colors) are jittered vertically while maintaining their linear position in the genome. Each locus is shown in the context of a 10 million bp window, with one additional inset for C. elegans showing a 200 kb window surrounding nrx-1. [file 12864_2020_6760_MOESM1_ESM.pdf]

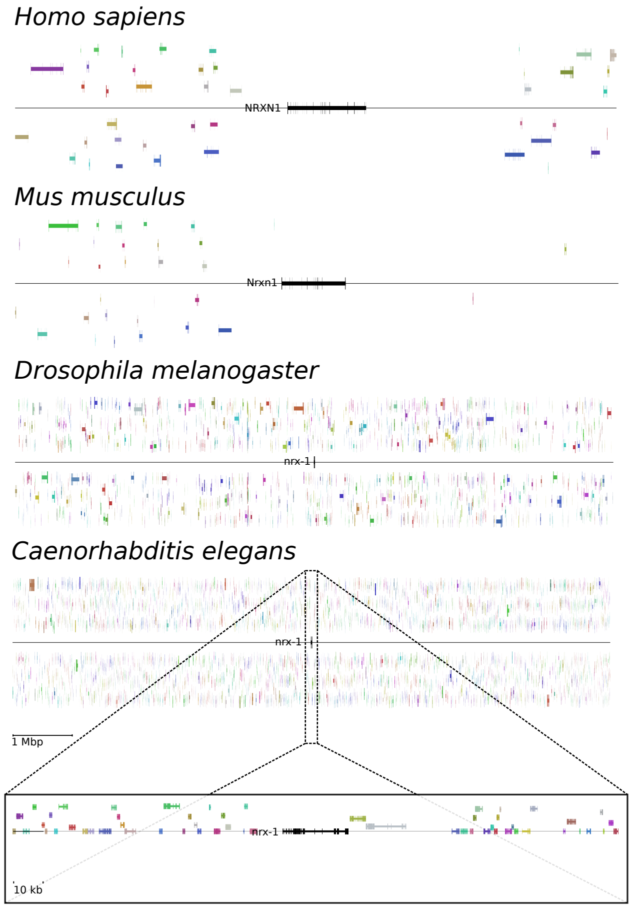


**Figure S1.** An exemplary gene size and density comparison at *NRXN1* ortholog loci. For each species, a *NRXN1* ortholog (black; *NRXN1* in *Homo sapiens*, *Nrxn1* in *Mus musculus*, and *nrx-1* in *Drosophila melanogaster* and *Caenorhabditis elegans*) is shown centrally, while other genes (random non-black colors) are jittered vertically while maintaining their linear position in the genome. Each locus is shown in the context of a 10 million bp window, with one additional inset for *C. elegans* showing a 200 kb window surrounding *nrx-1*.
