# Supplementary material for: Intron and gene size expansion during nervous system evolution
Source: BMC Genomics. 2020 May 14;21:360. doi: 10.1186/s12864-020-6760-4 (PMC7222433; doi:10.1186/s12864-020-6760-4)
Supplement: Supplementary file 3 — Additional file 3: Figure S2. Intron length versus ordinal position for genes with greater than 2-fold tissue enrichment. [file 12864_2020_6760_MOESM3_ESM.pdf]

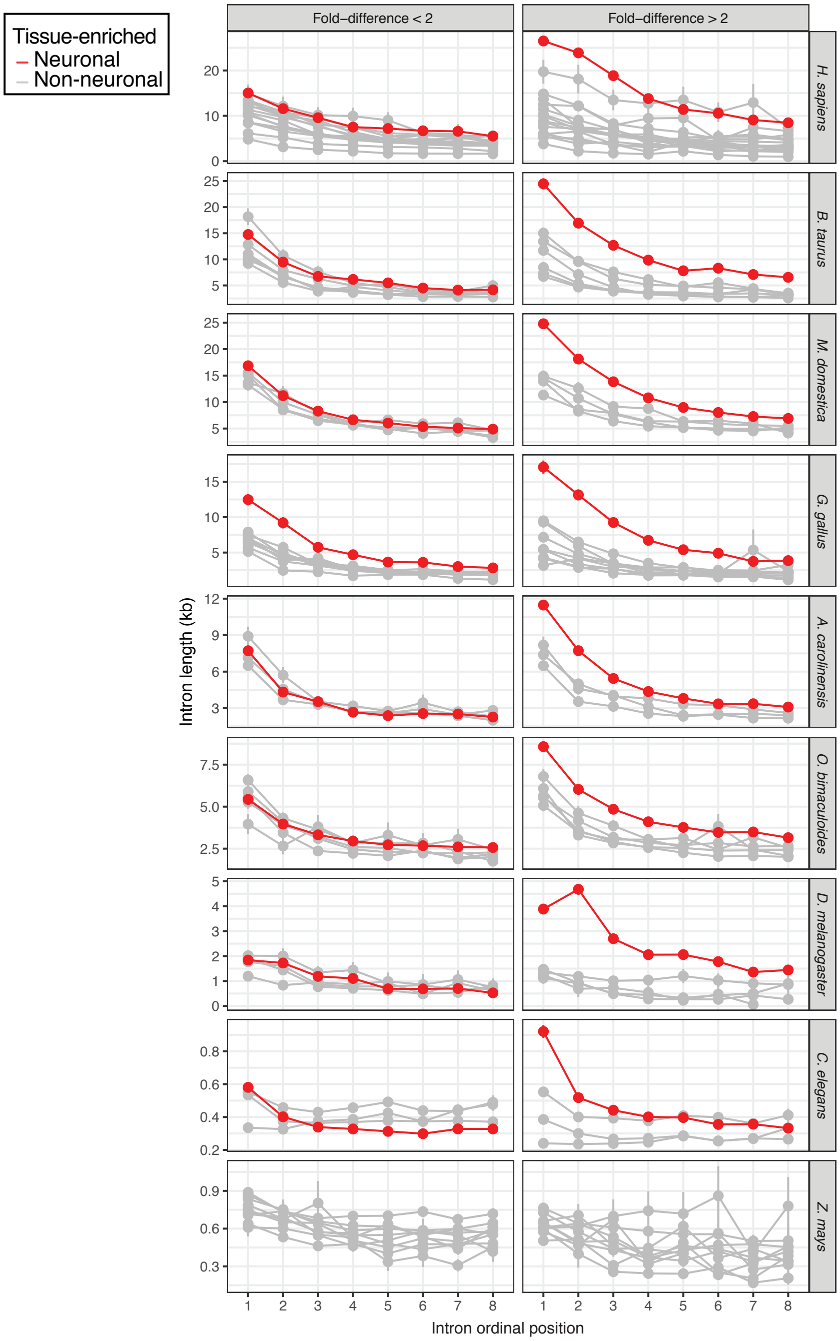

Figure S2. Intron length versus ordinal position for genes with greater than 2-fold tissue enrichment.
