# Supplementary material for: Intron and gene size expansion during nervous system evolution
Source: BMC Genomics. 2020 May 14;21:360. doi: 10.1186/s12864-020-6760-4 (PMC7222433; doi:10.1186/s12864-020-6760-4)
Supplement: Supplementary file 4 — Additional file 4: Figure S3. Non-smooth-quantile-normalized gene expression conditional on gene length. Each plot shows transcriptome data across multiple tissues from species presented in Fig. 1. Neuronal tissues or cells are shown in red and all other tissues and cell types are shown in grey, and transparent ribbons show 95% confidence intervals. (A) Genes were segregated into 100 bins according to gene length (kb), and points show average gene length of each bin. (B) Loess smoothing of gene expression conditional on gene length (kb). (C) Generalized additive model of gene expression conditional on gene length (kb). [file 12864_2020_6760_MOESM4_ESM.pdf]

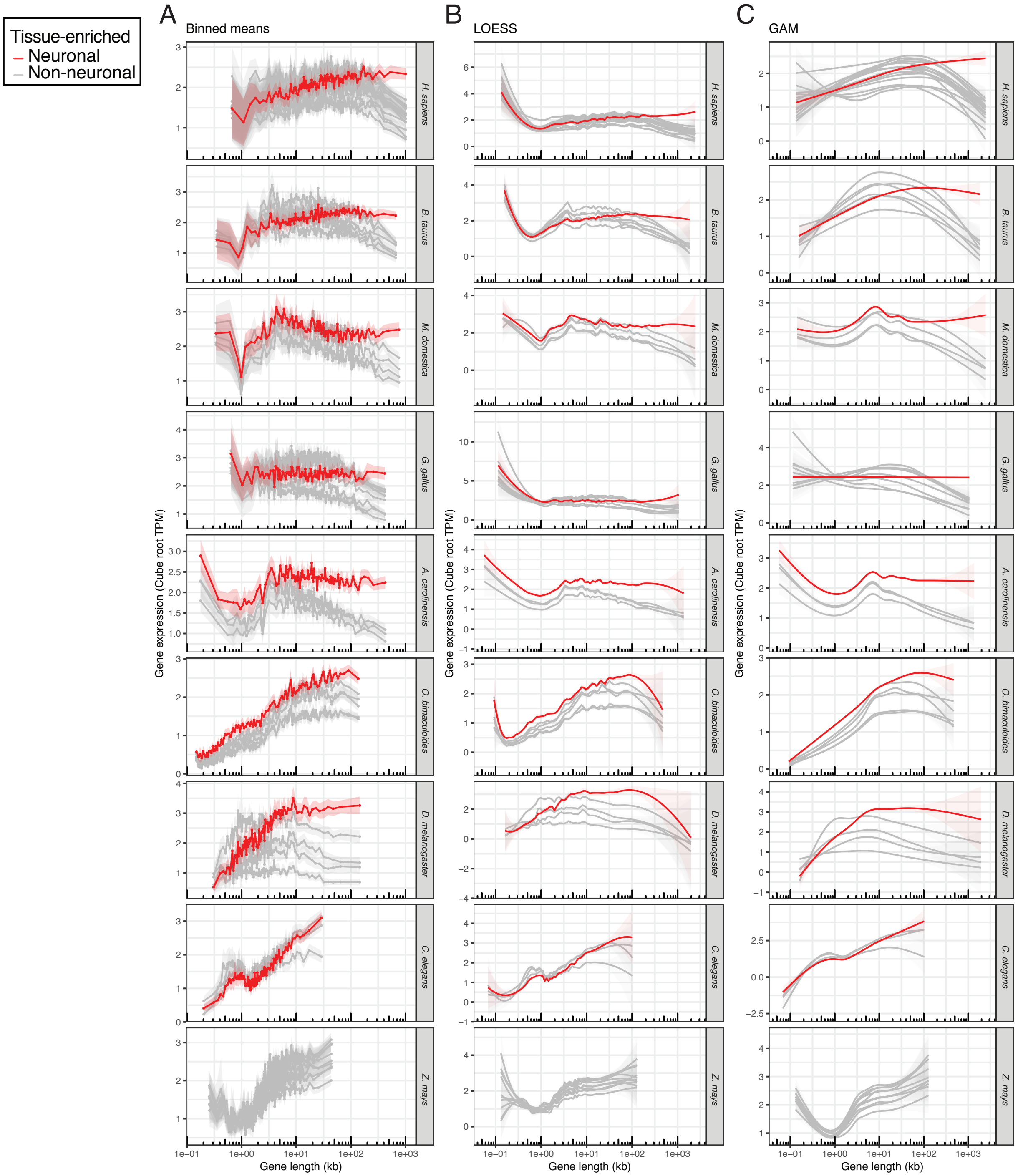

Figure S3. Non-smooth-quantile-normalized gene expression conditional on gene length. Each plot shows transcriptome data across multiple tissues from species presented in Fig. 1. Neuronal tissues or cells are shown in red and all other tissues and cell types are shown in grey, and transparent ribbons show 95% confidence intervals. (A) Genes were segregated into 100 bins according to gene length (kb), and points show average gene length of each bin. (B) Loess smoothing of gene expression conditional on gene length (kb). (C) Generalized additive model of gene expression conditional on gene length (kb).
