# Supplementary material for: Intron and gene size expansion during nervous system evolution
Source: BMC Genomics. 2020 May 14;21:360. doi: 10.1186/s12864-020-6760-4 (PMC7222433; doi:10.1186/s12864-020-6760-4)
Supplement: Supplementary file 5 — Additional file 5: Figure S4. Non-smooth-quantile-normalized gene expression conditional on gene length quantiles. Each plot shows transcriptome data across multiple tissues from species presented in Fig. 1. Neuronal tissues or cells are shown in red and all other tissues and cell types are shown in grey, and transparent ribbons show 95% confidence intervals. Gene lengths are shown as quantiles (100 bins). (A) Genes were segregated into 100 bins according to gene length, and points show average gene length of each bin. (B) Loess smoothing of gene expression conditional on gene length quantiles. (C) Generalized additive model of gene expression conditional on gene length quantiles. [file 12864_2020_6760_MOESM5_ESM.pdf]

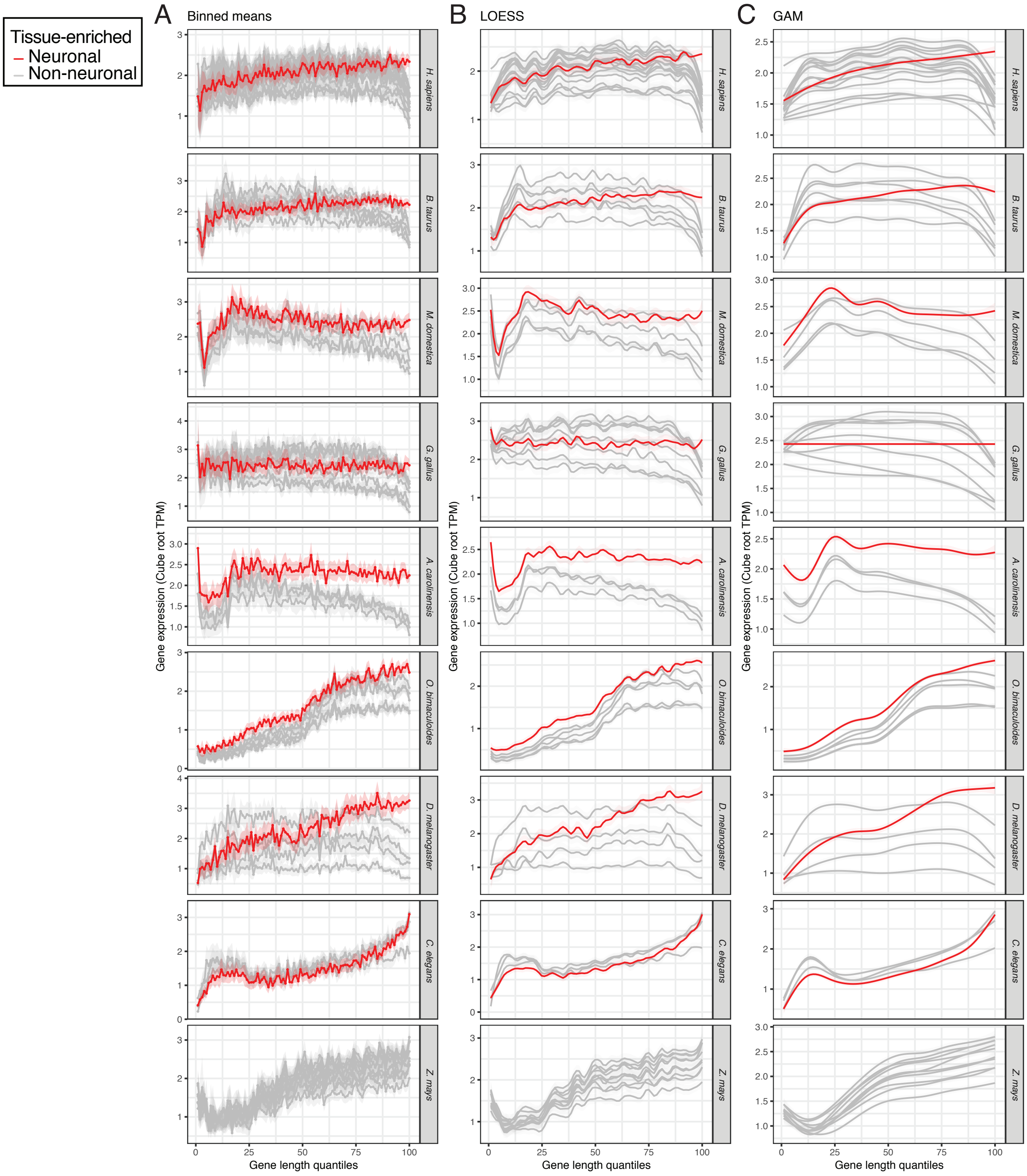

Figure S4. Non-smooth-quantile-normalized gene expression conditional on gene length quantiles. Each plot shows transcriptome data across multiple tissues from species presented in Fig. 1. Neuronal tissues or cells are shown in red and all other tissues and cell types are shown in grey, and transparent ribbons show 95% confidence intervals. Gene lengths are shown as quantiles (100 bins). (A) Genes were segregated into 100 bins according to gene length, and points show average gene length of each bin. (B) Loess smoothing of gene expression conditional on gene length quantiles. (C) Generalized additive model of gene expression conditional on gene length quantiles.
