# Supplementary material for: Intron and gene size expansion during nervous system evolution
Source: BMC Genomics. 2020 May 14;21:360. doi: 10.1186/s12864-020-6760-4 (PMC7222433; doi:10.1186/s12864-020-6760-4)
Supplement: Supplementary file 8 — Additional file 8: Figure S7. Gene features across clades. Joy plots showing distribution of gene features (median gene length, median intron length, median exon length, median number of exons per gene, and number of genes). [file 12864_2020_6760_MOESM8_ESM.pdf]

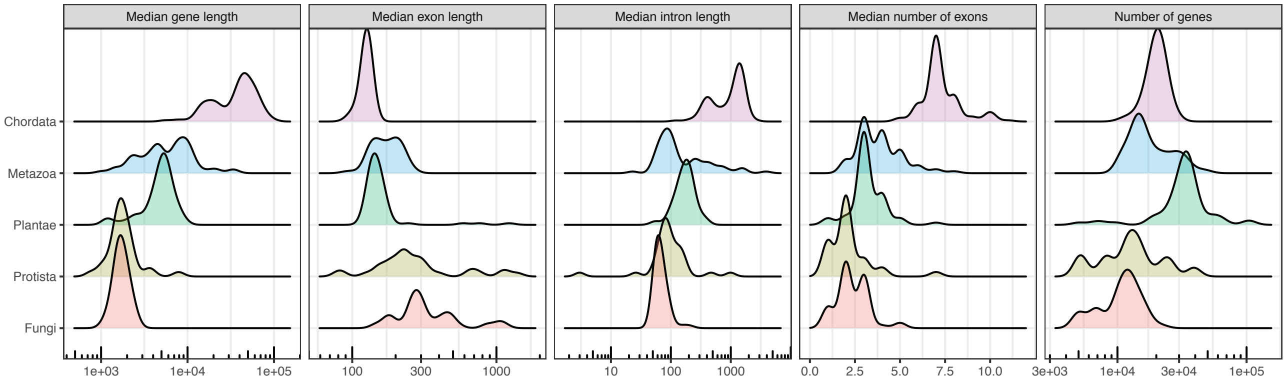

Figure S7. Gene features across clades. Joy plots showing distribution of gene features (median gene length, median intron length, median exon length, median number of exons per gene, and number of genes).
